# Visual word learning in adults with dyslexia

**DOI:** 10.3389/fnhum.2014.00264

**Published:** 2014-05-06

**Authors:** Rosa K. W. Kwok, Andrew W. Ellis

**Affiliations:** Department of Psychology, University of YorkYork, UK

**Keywords:** word learning, reading, dyslexia, word length, repetition, working memory, phonological awareness

## Abstract

We investigated word learning in university and college students with a diagnosis of dyslexia and in typically-reading controls. Participants read aloud short (4-letter) and longer (7-letter) nonwords as quickly as possible. The nonwords were repeated across 10 blocks, using a different random order in each block. Participants returned 7 days later and repeated the experiment. Accuracy was high in both groups. The dyslexics were substantially slower than the controls at reading the nonwords throughout the experiment. They also showed a larger length effect, indicating less effective decoding skills. Learning was demonstrated by faster reading of the nonwords across repeated presentations and by a reduction in the difference in reading speeds between shorter and longer nonwords. The dyslexics required more presentations of the nonwords before the length effect became non-significant, only showing convergence in reaction times between shorter and longer items in the second testing session where controls achieved convergence part-way through the first session. Participants also completed a psychological test battery assessing reading and spelling, vocabulary, phonological awareness, working memory, nonverbal ability and motor speed. The dyslexics performed at a similar level to the controls on nonverbal ability but significantly less well on all the other measures. Regression analyses found that decoding ability, measured as the speed of reading aloud nonwords when they were presented for the first time, was predicted by a composite of word reading and spelling scores (“literacy”). Word learning was assessed in terms of the improvement in naming speeds over 10 blocks of training. Learning was predicted by vocabulary and working memory scores, but not by literacy, phonological awareness, nonverbal ability or motor speed. The results show that young dyslexic adults have problems both in pronouncing novel words and in learning new written words.

## Introduction

The problems that dyslexic children and adults experience in reading and spelling have been well documented, even if there is continuing debate about the underlying causes of those difficulties (Snowling, 2001; Vellutino et al., 2004; Van den Broeck and Geudens, 2012). One aspect of reading skill that has received less attention than most in the literature, however, is how dyslexics learn new written words and how their ability to learn new words compares with that of normal readers (Reitsma, 1983; Ehri and Saltmarsh, 1995; Mayringer and Wimmer, 2000; Share and Shalev, 2004; Thomson and Goswami, 2010; De Jong and Messbauer, 2011). The current paper develops a methodology for studying basic aspects of word learning that we believe has considerable potential and applies it to understanding visual word learning in groups of dyslexic adults and normally-reading controls.

As children grow older, reading becomes an important source of new words that they must learn to recognize and understand if they are to function effectively (Cunningham et al., 2002; Cunningham, 2006; Nation, 2008, 2009). Nowhere is this more true than in higher education where, if students are to progress satisfactorily, they must learn new words connected with their academic studies that are often encountered first in written form (Mortimore and Crozier, 2006). Our concern in the present study is not with how dyslexics learn to associate new words with meanings, but rather with the process by which initially unfamiliar words become familiar through exposure and repetition, reaching the point where they can be recognized and processed as whole units rather than in piecemeal fashion.

The starting point for our investigation was a study by Weekes (1997) who asked skilled adult readers (undergraduate students at a UK university) to read aloud a mixture of familiar words and invented nonwords as quickly as possible. Naming latencies were measured as the time between a word or nonword appearing on the screen and the participant beginning to pronounce it. The words were either high frequency (e.g., bed, large) or low frequency (e.g., beg, latch): the nonwords were pronounceable sequences of letters that could be words but happen not to be (e.g., bam, lorge). Words and nonwords varied in length from 3 to 6 letters. In line with previous studies, naming latencies were substantially slower for the nonwords than for the familiar words (cf. Lupker et al., 1997; Rastle et al., 2003). Latencies for the nonwords increased substantially as letter length increased. In contrast, low frequency words showed only a small effect of length on naming speeds while high frequency words showed no significant effect at all. Stronger effects of length on naming latencies for nonwords than words in skilled readers have now been reported in English, German and French (Ziegler et al., 2001; Juphard et al., 2004; Valdois et al., 2006) while stronger effects of length on latencies for low than high frequency English words have been reported by Yap and Balota (2009) and others.

Weekes (1997) argued that slower reading of nonwords than familiar words, and larger effects of letter length for nonwords than words, could be explained within the dual-route (DRC) model of visual word recognition proposed by Coltheart et al. (2001). According to the DRC model, when an unfamiliar word or nonword is encountered for the first time, it is translated from written into spoken form through the application of letter-sound (grapheme-phoneme) conversion rules (referred to in the DRC model as the *nonlexical route*). The grapheme-phoneme conversion rules act in a serial, left-to-right manner, working systematically through a novel word from the beginning to the end until a pronunciation has been generated (Coltheart and Rastle, 1994). As a new word becomes familiar through repeated encounters, entries are created for that word in the mental lexicon. In the DRC model that process of lexicalization involves creating a representation of the written form of the word in an *orthographic input lexicon* and a representation of its spoken form in a *phonological output lexicon*. A route from print to sound becomes available for the newly-learned word through the two lexicons. This is known as the *lexical route*. Access to the orthographic input lexicon for familiar written words is both fast and parallel, with all of the component letters in a word being processed simultaneously. As a result, pronouncing a familiar word (lexical route) is faster than generating the pronunciation of an unfamiliar word or nonword (nonlexical route) and the impact of letter length is greatly reduced in familiar words (see Coltheart et al., 2001, pp. 238–239, where a simulation of the Weekes, 1997, results is presented). The more familiar a word is, the more its pronunciation will be captured by the lexical route, hence the progressively smaller effect of length seen in low and high frequency words.

If this account is broadly correct, it should be possible to observe the transition from nonlexical to lexical reading by presenting unfamiliar words or nonwords repeatedly. When the novel items are read for the first time, naming should reflect the operation of the nonlexical route: latencies should be slow and sensitive to the number of letters in the sequence. But as the novel words become familiar, lexical representation should be established and processing should make the transition from nonlexical to lexical reading, with naming latencies becoming become faster and less affected by length. Maloney et al. (2009) observed the beginnings of this transition. They took the 100 nonwords varying in length from 3 to 6 letters that were used by Weekes (1997) and presented them to skilled readers in four consecutive blocks of trials. Participants were instructed to read each one aloud as quickly as possible. As predicted, naming latencies became faster across the four blocks as the items became more familiar and the effect of length reduced.

In unpublished experiments we have replicated and extended Maloney et al.'s (2009) results. In one experiment we measured naming latencies for 4-letter, single-syllable nonwords and 7-letter, two-syllable nonwords. The nonwords were presented 10 times in consecutive blocks of trials, using a different random order of presentation in each block. Accuracy was very high across the experiment. In the first block, when all of the nonwords were new and unfamiliar, naming latencies were relatively slow and the effect of length was substantial. Reaction times (RTs) then reduced with repeated presentations and the impact of length diminished, becoming non-significant after five or six presentations of the nonwords. We obtained the same pattern of results in a second experiment using a different set of nonwords. In that experiment we also invited the participants back for a second testing session 7 days after the first session to assess the extent to which the learning effects persisted in the absence of any further experience with the nonwords. Naming latencies in block 1 of day 7 were a little slower than at the end of day 1, but much faster than at the start of day 1, demonstrating considerable retention of lexical knowledge about the newly-learned items. By the fourth block of day 7, the effect of length had completely disappeared: the nonwords had become familiar, created lexical entries, and been unitized to the point where they were read aloud in the same way as familiar words.

The present paper compares the performance of university and college students with a diagnosis of dyslexia with typically-reading controls on the same task. Nonwords composed of either 4 or 7 letters were presented 10 times in a first testing session, then 10 more times in a second testing session 7 days later. Accuracy of reading the nonwords aloud was assessed along with naming latencies. Bruck (1990) and Ben-Dror et al. (1991) found slower and less accurate reading of both words in nonwords in American college dyslexics than controls. Similar results have been reported for Polish (Reid et al., 2006) and Swedish (Wolff, 2009) dyslexic university students and controls. Less accurate reading aloud of both words and nonwords by student dyslexics than controls was reported by Snowling et al. (1997) and Hatcher et al. (2002) in very similar participant groups to those reported here (see also Callens et al., 2012; Deacon et al., 2012). These observations, combined with reports of less proficient reading of both nonwords and words by dyslexic children (Zoccolotti et al., 2005; Reid et al., 2006; Wolff, 2009; Paizi et al., 2013), led us to expect that the dyslexic students in our experiment would be slower and possibly less accurate than controls throughout the experiment, not only when the nonwords were presented for the first time, but even after multiple encounters.

We also expected that the adult dyslexics would show stronger effects of letter length on reading speed than the controls. There are two reasons why such a difference could come about. First, it has often been proposed that nonword reading presents particular problems for dyslexics (Rack et al., 1992; Herrman et al., 2006; though see Van den Broeck and Geudens, 2012). Wimmer (1996), for example, found that 10-year-old German dyslexic children read nonwords more slowly than younger normal readers who were matched to the dyslexics on the speed of reading familiar, high frequency words. If nonlexical reading is indeed differentially poor in many dyslexics, length effects should be greater in dyslexics than typical readers because the dyslexics will require more time per additional letter to convert that letter into sound.

Second, if dyslexics are slower than typical readers to create new lexical entries, then in the course of an experiment involving 20 presentations of each nonword across two separate sessions, the dyslexics may be slower than the controls to create orthographic and phonological representations for the novel items. The result would be that they spend more time reading nonlexically (with consequent length effects) and would be slower to switch to lexical reading (with reduced length effects). We are not aware of any studies of word learning in dyslexia that have involved adult participants, but research involving dyslexic children suggests problems learning both the spoken and the written forms of new words. Regarding the learning of spoken word-forms, Mayringer and Wimmer (2000) found that German-speaking dyslexic children were impaired at learning novel spoken words that were taught as the names of children shown in pictures. In contrast, the dyslexics were unimpaired at learning to associate familiar German names with pictures of children. The authors concluded from this that the dyslexic children's difficulty lay in learning the new spoken words rather than in associating names with people (see also Elbro and Jensen, 2005; Thomson and Goswami, 2010).

Mayringer and Wimmer (2000) suggested that if dyslexics have problems learning new written words, part of those problems could lie in learning the spoken (phonological) forms rather than their written (orthographic) forms. Visual word learning involves creating phonological as well as orthographic representations: difficulties in learning spoken word-forms would be expected to impact on visual word learning. The few published studies of visual (rather than spoken) word learning in dyslexia suggest, however, that dyslexics have problems learning new written word-forms over and above any problems they experience in learning spoken words (Reitsma, 1983; Ehri and Saltmarsh, 1995; Share and Shalev, 2004; De Jong and Messbauer, 2011; O'Brien et al., 2013). Reitsma (1983; Expt. 3) compared visual word learning in Dutch children with reading disabilities with learning in a group of younger normal readers. The children first practiced reading aloud novel words embedded in sentences. Three days later they were asked to read aloud the novel words as quickly as possible as they were presented individually on a computer screen. Half of the novel words were presented in exactly the same written form as in the training while the other half were presented in a form that had a different spelling but was pronounced the same. (An equivalent English example might be to train children to read *breet* then test them three days later on either *breet* or *breat*). The normal readers were faster to read aloud the versions of the novel words that they had been trained on three days earlier than the re-spelled version, though they were faster on both than on entirely new and untrained nonwords (so faster on *breet* than *breat* but faster on both of them than on *broat*). In contrast, the children with reading disability read both forms of the trained novel words (*breet* and *breat*) faster than the untrained items (*broat*) but showed no difference between the versions of the trained items that preserved the original spellings (*breet*) and the versions that changed those spellings (*breat*). The implication of these results is that the normal readers learned both the orthographic and phonological forms of the novel words in training and retained that knowledge through to the test three days later. The disabled readers remembered something of the phonological forms of the trained novel items across the retention interval but seemed not to retain any detectable orthographic information.

If dyslexic children combine less efficient nonlexical reading with slower creation of lexical entries, we would expect them to show larger length effects in nonword reading than typically-reading controls. We would also expect dyslexics to show larger effects of letter length in word reading arising from the fact that they are less efficient than controls at switching from nonlexical to lexical reading so read more words nonlexically than controls do. This prediction is supported by reports of stronger effects of letter length on naming latencies for real words in dyslexic children than controls in English, Dutch, German, Spanish and Italian (e.g., Ziegler et al., 2003; Marinus and De Jong, 2010; Paizi et al., 2011; Davies et al., 2013; Martelli et al., 2014).

Dyslexics may have difficulty learning new spoken and written word-forms but dyslexic Italian children have been reported to read words faster than nonwords (Paizi et al., 2013) thereby demonstrating some acquisition of word-specific knowledge. Paizi et al. (2013) also reported faster reading of high than low frequency words in dyslexic Italian children, indicating that regular exposure facilitates the creation of effective lexical entries in those readers. If dyslexics are capable of building up a vocabulary of words they can read in a relatively wholistic manner, albeit more slowly and effortfully than typical readers, that could explain the reduction in the impact of letter length on word reading with age that Zoccolotti et al. (2005) and De Luca et al. (2008) observed in both dyslexic Italian children and controls. Hence, on the basis of this admittedly incomplete literature, much of which is concerned with children rather than adults, we expected to see signs of word learning in the dyslexic participants in our experiment (i.e., faster naming latencies across blocks and a reduction in the impact of letter length with repeated exposure). We expected, however, that word learning would occur more slowly in the dyslexic participants than in controls (typical readers) and that if convergence between reading speeds for shorter and longer items was achieved, it would require more presentations of the nonwords.

Finally, our participants were given a short battery of tests to characterize their broader cognitive abilities. The cognitive profiles of dyslexic students at the same institution as many of the participants in the present study (the University of York, UK) were described a decade ago by Hatcher et al. (2002) and more recently by Warmington et al. (2013b). Hatcher et al. (2002) found that the student dyslexics performed at comparable levels to normally-reading controls on nonverbal ability (Raven's Advanced Progressive Matrices) but more poorly on a range of measures including verbal ability (WAIS-R vocabulary), word reading and spelling, forward and backward digit span, phonological tasks [object naming, digit naming and spoonerisms (exchanging sounds between words)] and mental arithmetic. Similar profiles were reported by Snowling et al. (1997) and Warmington et al. (2013b) for UK student dyslexics and Callens et al. (2012) for Belgian dyslexic students. A wider review and meta-analysis of dyslexia in adults is provided by Swanson and Hsieh (2009).

In addition to comparing the dyslexics and controls on the test battery, we used regression analyses to explore the ability of performance on the different cognitive tests to predict two aspects of performance in the experiment, namely initial reading speeds for the longer (7-letter) nonwords and the change in reading speeds across the 10 presentations in the first testing session. Initial reading speeds assess efficiency of converting unfamiliar letter sequences into sounds (in DRC terms, the efficiency of the nonlexical route), while the change in RTs across repetitions assesses the efficiency of word learning and the switch from nonlexical to lexical reading. Previous research has associated the speed and accuracy of reading nonwords or unfamiliar words with phonological awareness (Durand et al., 2005; Melby-Lervåg et al., 2012). For example, Pennington et al. (1990) documented persisting deficits in phonological awareness in adult dyslexics that were particularly linked to problems with nonword reading. Training studies have suggested, however, that phonological awareness must be linked to a knowledge of how letters map onto phonemes if improvements in phonological awareness are to be translated into improvements in reading (Hatcher et al., 1994; Melby-Lervåg et al., 2012).

Word learning has been more strongly associated with working memory than with phonological awareness (Gathercole et al., 1997, 1999; Avons et al., 1998). For example, Gathercole et al. (1999) reported an association between phonological working memory and vocabulary size in both 4-year-old and teenage children. Experimental studies by Jarrold et al. (2009) and Majerus and Boukebza (2013) reported a relationship between verbal working memory and ability to learn the form (rather than the referent) of new words by children and teenagers while Martin and Ellis (2012) found that word learning in an artificial second language by university students was predicted by performance on phonological short-term / working memory taks. Short-term and working memory have consistently been found to be impaired in dyslexia (Swanson et al., 2009) which may relate to the problems in word learning mentioned above.

## Materials and methods

### Participants

Participants were 30 students with a diagnosis of dyslexia (20 female, 10 male) and 30 typical readers who served as a control group (12 female, 18 male). The dyslexic students had a mean age of 21.5 years (*SD* = 3.6; range 17–36) while the controls had a mean age of 20.7 years (*SD* = 3.2; range 17–32). All were native speakers of English with normal or corrected-to-normal vision. The participants were students at the University of York (*n* = 27 per group), York Saint John University (*n* = 1 per group) and York College (*n* = 2 per group). The participants with dyslexia had all been diagnosed by a registered educational psychologist and supplied a copy of their diagnosis documents to the experimenters. Individuals with additional learning disabilities, a history of mental illness, epilepsy or other neurological disorders were excluded. Participants received either course credit or a small payment. The experiment was approved by the Ethics Committee of the Department of Psychology, University of York.

### Test battery

The psychological test battery given to all the participants contained tests assessing vocabulary, reading and spelling, phonological awareness, working memory, nonverbal ability and motor speed. Published tests were scored according to the test manuals and the results are presented as standardized scores.

#### Vocabulary

Vocabulary was assessed using the Vocabulary subtest of the WASI which requires participants to define words verbally.

#### Word reading

Word reading was assessed using the reading subtest of the Wide Range Achievement Test (WRAT 4; Wilkinson and Robertson, 2006) which involves reading aloud single words of increasing length and difficulty (from see to synecdoche) and the Sight Word Efficiency subtest of the Test of Word Reading Efficiency (TOWRE SWE; Torgesen et al., 1999) which requires participants to read aloud as many words of increasing length and difficulty as possible in 45 s.

#### Nonword reading

Nonword reading was assessed using the Phonemic Decoding Efficiency (PDE) subtest of the TOWRE which requires participants to read aloud as many nonwords of increasing length and difficulty as possible in 45 s.

#### Word spelling

Word spelling was assessed using the Spelling Subtest of the WRAT 4 which requires participants to write single words to dictation.

#### Phonological awareness

Phonological awareness was measured using that part of the elision test from the Comprehensive Test of Phonological Processing (CTOPP; Wagner et al., 1999) in which a single initial, medial or final phoneme of a word must be deleted and the participant must say what remains (e.g., deleting the /k/ from “fixed” and responding “fist”).

#### Working memory

Working memory was assessed using four tests from the Automated Working Memory Assessment (AWMA; Alloway, 2007). All the tests used span procedures in which sequence lengths were increased to the point where three or more errors were made within a block of trials. Standardized scores were calculated for each test. *Verbal short-term memory* was measured using immediate serial recall of lists of digits presented auditorily at a rate of 1/s. *Verbal working memory* was assessed using a test in which participants were presented with a sequence of spoken sentences. They were required to decide whether each sentence was true or false then recall the final words of each of the sentences at the end of the sequence. *Visuospatial short-term memory* was assessed using a dot matrix task in which a sequence of red dots appeared in squares of a 4 × 4 grid at a rate of one per 2 s. At the end of the sequence, the participant was required to touch the squares of the grid in the same order. *Visuospatial working memory* was measured using a spatial recall task. Participants were presented with pairs of shapes. The shape on the right always had a red dot in it. The shape on the left was either the same as the one on the right or different. The shape on the left could also be rotated with respect to the one on the right. The participant's task was first to say whether the two shapes were the same or different. After making those judgments to a sequence of pairs of shapes, the participant then had to indicate in the correct order where the red dot was positioned in each of the shapes on the right using a compass display with three points.

#### Nonverbal ability

Nonverbal ability was assessed using the matrix reasoning subtest of the Wechsler Abbreviated Scale of Intelligence (WASI; Wechsler, 1999).

#### Motor speed

Motor speed was assessed using a set of tapping tasks (Warmington et al., 2013a). Participants were asked to tap keys on a computer keyboard as many times as possible within 5 s. The start and end of each time interval was signaled both visually and auditory. The task consisted of three conditions with 6 trials in each condition. In Condition 1, the participants tapped one key using the index finger of their preferred hand as many times as possible. In Condition 2, the participants alternately tapped two keys using the index finger of their preferred hand as many times as possible. In Condition 3, the participants alternately tapped two keys using the first two fingers of their preferred hand as many times as possible. The score is the average time between taps across the three conditions.

### Experimental stimuli

The experimental stimuli were 12 4-letter, single-syllable nonwords and 12 7-letter, two-syllable nonwords. To reduce problems of voice key activation, none of the stimuli began with a voiceless fricative (“f,” “s,” “sh,” or “th”). The 4- and 7-letter items were matched on naming accuracy from a pilot study involving typical student readers. They were also matched on mean log bigram frequency (4-letter mean = 3.28, range 2.72–3.57; 7-letter mean = 3.27, range 3.10–3.43; Duyck et al., 2004) and on initial letters and phonemes. The 4-letter experimental nonwords were: *brup, carg, dreb, jeph, lont, munt, nate, plin, relb, trok, varb*, and *zort*. The 7-letter experimental nonwords were: *blispod, coftrip, drentcy, joshule, larquof, mattoch, nelpoon, pronnet, roffler, trimsol, vushood*, and *zadroon*. Sixteen additional nonwords (8 4-letter and 8 7-letter) were selected for use in practice trials prior to the main experiment.

### Procedure

Participants attended for two sessions. The first session began with the participants reading and signing a consent form then completing the psychological assessment battery. That took approximately 45 min. After a break of around 10 min they began the experimental task. They were given practice at reading aloud 8 4-letter and 8 7-letter nonwords presented in a random order. That was followed by the 10 blocks of the experiment. Participants were seated approximately 60 cm from a computer screen on which the nonwords were displayed in black, lower case letters on a white background. The nonwords were presented in 18-point Times New Roman font with a height on the screen of approximately 10 mm. Each trial consisted of a centrally-presented fixation cross displayed for 1000 ms, followed by the nonword stimulus for 2000 ms, then a blank screen for 1000 ms before the next trial began. Participants were instructed to read each nonword aloud as quickly and as accurately as possible. The 24 nonwords were presented once in a random order. Participants were informed when the block was complete and pressed the space bar on a computer keyboard to initiate the next block when they were ready to continue. This process was repeated across 10 blocks with the stimuli being presented in a different random order in each block. Participants wore headphones with a high-sensitivity microphone connected to a voice key that was linked to the computer. Presentation of the stimuli and recording of naming latencies was controlled by E-prime experiment generator software (version 1.2; Schneider et al., 2002). The experimenter noted any trials in which the participant misread a nonword, hesitated or made a false start or other form of error.

Participants returned 7 days later for the second session which was a repeat of session 2 involving reading all the experimental nonwords aloud 10 more times in 10 blocks using a different random order in each block.

## Results

### Performance on the test battery

Table 1 shows the results for the dyslexics and controls on the battery of tests together with the results of *t*-tests comparing the two groups along with the effect sizes (*r*; Field, 2009). Dyslexics performed significantly less well than the controls on every test except nonverbal reasoning. The effect sizes for the differences between the groups were largest for nonword reading, followed by spelling and word reading. The effect sizes for the differences between groups on verbal and visuospatial working memory tasks were similar.

**Table 1:** **Results of the dyslexic and typical readers on the psychological test battery**.

	**Dyslexics**		**Typical readers**		***t*-tests and effect sizes (*r*)**
	**Mean**	***SD***	**Mean**	***SD***	
**VOCABULARY**					
WASI Vocabulary	56.50	7.68	63.73	6.78	*t*_(58)_ = 3.87, *p* < 0.001; *r* = 0.45
**WORD READING**					
WRAT 4 Reading	99.00	7.44	117.30	12.80	*t*_(58)_ = 6.77, *p* < 0.001; *r* = 0.66
TOWRE-SWE	82.00	11.03	97.44	10.68	*t*_(58)_ = 7.21, *p* < 0.001; *r* = 0.69
**NONWORD READING**					
TOWRE-PDE	86.63	10.23	108.08	7.72	*t*_(58)_ = 12.01, *p* < 0.001; *r* = 0.84
**SPELLING**					
WRAT 4 Spelling	96.50	12.35	121.33	11.86	*t*_(58)_ = 7.95, *p* < 0.001; *r* = 0.72
**PHONOLOGICAL AWARENESS**					
CTOPP Elision	7.27	1.76	9.00	1.68	*t*_(58)_ = 3.40, *p* < 0.001; *r* = 0.41
**WORKING MEMORY**					
AWMA verbal STM	87.67	12.82	101.53	14.54	*t*_(58)_ = 3.92, *p* < 0.001; *r* = 0.46
AWMA verbal WM	93.00	13.86	105.97	14.55	*t*_(58)_ = 3.53, *p* = 0.001; *r* = 0.42
AWMA visuospatial STM	90.33	11.56	108.83	13.05	*t*_(58)_ = 5.81, *p* < 0.001; *r* = 0.61
AWMA visuospatial WM	95.87	16.09	106.89	11.92	*t*_(58)_ = 3.02, *p* < 0.01; *r* = 0.37
**NONVERBAL ABILITY**					
WASI Matrix reasoning	54.60	7.75	55.77	5.73	*t*_(58)_ = 0.66, *p* = 0.510; *r* = 0.09
**Motor speed**	267.66	55.54	224.26	35.11	*t*_(58)_ = −3.54, *p* = 0.001; *r* = 0.42

### Performance on the experimental task

Naming errors, hesitations and failures to activate the voice key were removed from the analysis of performance on the experimental task along with RTs less than 100 ms or longer than 2.5 *SD*s above the mean (defined separately for each participant in each block and for each length). Table S1 (Supplementary Materials) shows the full results (accuracy and mean RTs for correct, trimmed responses). Accuracy was very high (97.3% correct overall and never less than 95.5% correct for either group in any condition or block of trials). Given the high levels of accuracy in both groups, nonparametric Mann-Whitney *U* tests found no significant difference between dylexics and typical readers on overall accuracy across the two days for either 4-letter nonwords, *U*_(60)_ = 464, *Z* = 0.208, *p* = 0.835, or 7-letter nonwords, *U*_(60)_ = 346, *Z* = −1.548, *p* = 0.122. Wilcoxon matched pairs, signed ranks tests found no difference between accuracy for 4- vs. 7-letter nonwords across the two sessions for both groups of participants combined, *W*_(12)_ = 23.0, *Z* = 1.26, *p* = 0.209.

#### Naming latencies (RTs)

**Figure 1:**
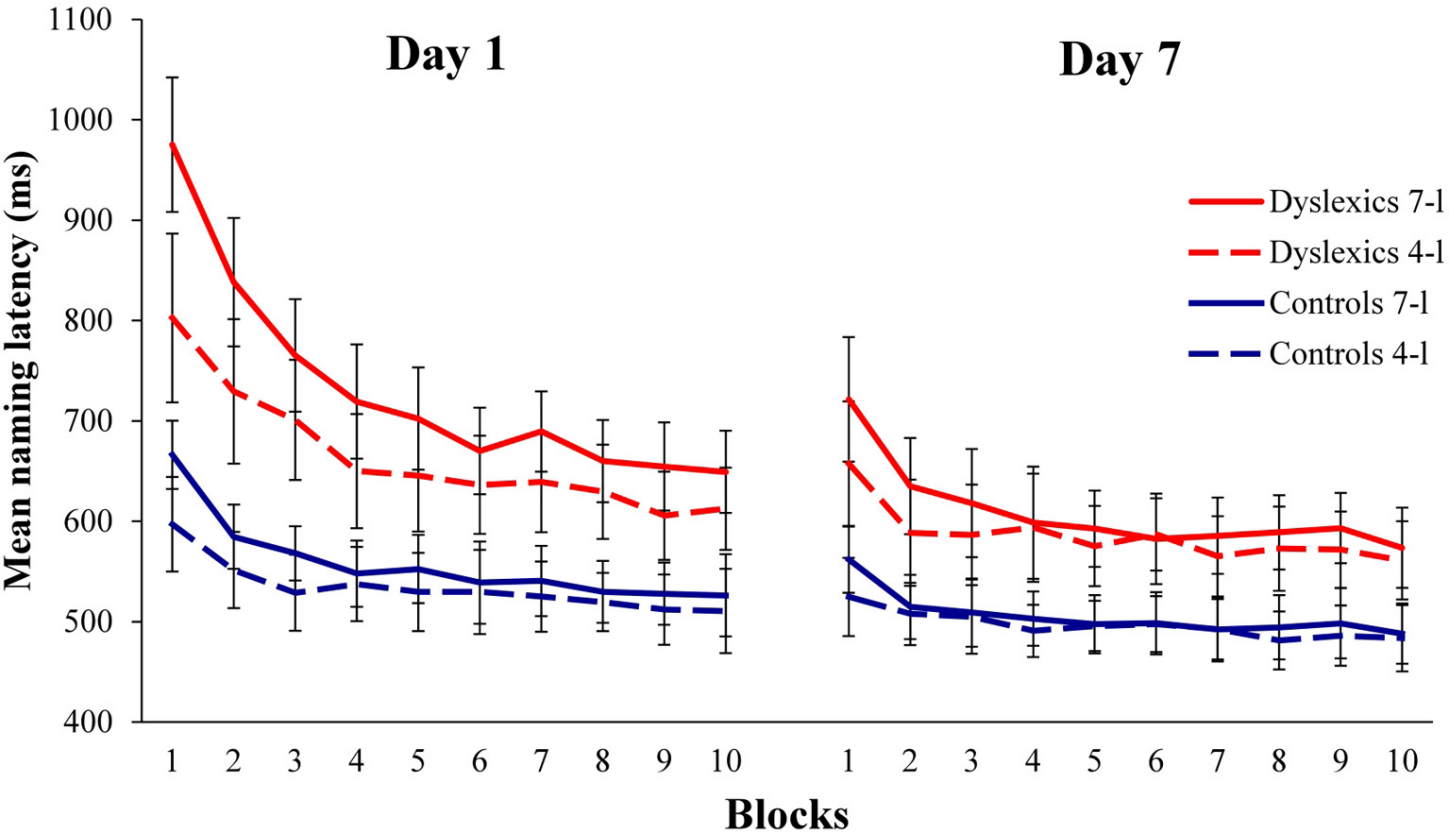
**Naming RTs to 4- and 7-letter nonwords in dyslexics and controls across two sessions (10 blocks per session)**. Error bars show 95% CIs.

The main analyses focused on the RT data from the experimental task. Figure 1 shows the pattern of RTs for correct, trimmed responses across blocks for the dyslexics (in red) and the controls (in blue). Inspection of the figure indicates that naming latencies were slower for the dyslexics than the controls throughout the experiment. At the start of the experiment, both groups were slower to read aloud 7- than 4-letter nonwords. The difference in naming RTs for shorter and longer nonwords reduced with repetitions, but the dyslexic participants appear to have required more exposures to the nonwords before the RTs for shorter and longer items converged. These indications were explored in a series of ANOVAs. When Mauchly's test of sphericity was significant, the Greenhouse-Geiger correction was applied. Full details of the statistical analyses are presented in the Appendix (Supplementary Materials) where effect sizes are reported in terms of the partial eta squared statistic (η^2^_*p*_). We will summarize the important outcomes here.

***Global analysis***. The first ANOVA was a global analysis conducted on the RT data for both testing sessions with Group, Day, Blocks and Length as factors. There were significant main effects of Group (faster overall RTs for the controls than the dyslexics), Day (faster RTs on day 7 than day 1), Blocks (RTs becoming faster across blocks) and Length (faster overall RTs to 4- than 7-letter nonwords). All of the interactions were significant, including the interaction between Group and Length (larger length effects in the dyslexics than the controls) and Groups × Blocks × Length (the reduction in the length effect across blocks occurring more quickly in the controls than in the dyslexics). These results were explored further by means of separate analyses of RTs in day 1 and day 7, including separate analyses of the performance of the dyslexic and control groups on each day.

***Day 1***. Day 1 RTs were analyzed with Group, Blocks and Length as factors. There were significant main effects of Group (faster RTs in the controls than the dyslexics), Blocks (RTs becoming faster across blocks) and Length (faster RTs to 4- than 7-letter nonwords). All of the interactions were significant. Day 1 RTs were then analyzed separately for controls and dyslexics. The controls showed significant main effects of Blocks and Length with a Blocks × Length interaction. Bonferroni-corrected *t*-tests were used to compare RTs to 4- and 7-letter nonwords in blocks 1–10. The effect of length was significant for the controls in blocks 1, 2, and 3 but was no longer significant from block 4 onwards. The dyslexics also showed effects of Blocks and Length combined with a Blocks × Length interaction. In their case, Bonferroni-corrected *t*-tests found effects of length in blocks 1–5, 7, 9, and 10 with marginally significant effects in blocks 6 and 8 (see Appendix; Supplementary material).

In sum, nonword naming RTs in day 1 were slower for the dyslexics than the controls. Both groups showed significant effects of length in the first three blocks, but while the controls showed no difference in naming speed after block 3, the dyslexics continued to show longer RTs to 7- than 4-letter nonwords throughout day 1.

***Day 7***. The next set of analyses focused on RTs in day 7. As in day 1, there were main effects of Group (faster RTs in the controls than the dyslexics), Blocks (RTs becoming faster across blocks) and Length (faster RTs to 4- than 7-letter nonwords). A significant Blocks × Length interaction reflected an overall reduction in the effect of length across blocks. There were also significant Group x Blocks and Group × Length interactions reflecting more change across blocks and stronger effects of length in the dyslexics than the controls. The 3-way Group × Blocks × Length interaction was marginally significant (*p* = 0.06). These interactions were explored further by means of separate analyses of day 7 RTs for controls and dyslexics.

Controls showed effects of Blocks and Length on day 7 with a significant Blocks × Length interaction. Bonferroni-corrected *t*-tests found a difference in RTs to 4- and 7-letter nonwords in block 1 only. Dyslexics also showed effects of Blocks and Length with a Blocks × Length interaction. In their case, Bonferroni-corrected *t*-tests found effects of length in blocks 1, 2, and 3, but not from block 4 onwards.

In sum, the controls showed a small effect of length at the start of day 7, but that effect disappeared by block 2. Dyslexics required 3 or 4 presentations in day 7 before they began to show (for the first time) no significant difference between naming RTs to short and long nonwords.

### Predictors of intial nonword reading speed and novel word learning

The final set of analyses brought together performance on the test battery with two aspects of the naming latency data. Nonlexical reading skill (decoding) was measured in terms of RTs to 7-letter nonwords in block 1 of day 1 while novel word learning was measured in terms of the change in RTs to 7-letter nonwords from block 1 to block 10 on day 1.

The number of predictor variables was reduced before the regression analyses were run, and some of the variables were transformed to improve the normality of their distributions. There were high correlations among the two word reading tests and the word spelling test (*r*s = 0.67–0.84, all *p*'s < 0.001). A composite Literacy score was therefore calculated for each participant by averaging the standardized scores from the WRAT Reading, TOWRE word reading and WRAT Spelling tests. To avoid using nonword reading in one task to predict nonword reading in another task, performance on the TORE-PDE nonword reading task was not included in the composite Literacy score. Substantial correlations were also observed among the four tests of working memory (*r*s = 0.50–0.56, all *p*'s < 0.001). A composite Working memory score was therefore computed for each participant by averaging the standardized scores from the four working memory tasks.

Univariate normality was tested for each predictor and the dependent variables (RTs to 7-letter nonwords in blocks 1 and 10 of day 1). Phonological awareness, Nonverbal ability and Motor speed were found to violate the assumption of normality (Kolmogorov-Smirmov test of normality, *p* < 0.05). Distributions approximated normality most closely when Phonological awareness was reverse transformed (thereby reversing the normal direction of correlations) and Nonverbal ability and Motor speed were square root transformed. RTs were log transformed to reduce skew.

Reducing the number of variables helps to reduce the risks associated with multicollinearity (intercorrelation among the predictor variables). Multicollinearity among the final versions of the predictor variables was assessed using the variance inflation factor (VIF). VIF scores of less than 4 indicate that the result will not significantly influence the stability of the parameter estimates (Myers, 1990). VIF scores for the predictor variables ranged between 1.04 and 3.01.

Table 2 shows the correlations among the final predictor variables; also the correlations between the predictor variables and RTs to 7-letter nonwords in block 1 of day 1. There were significant correlations among all the predictor variables except Nonverbal ability which did not correlate significantly with any of the other predictors. All of the predictors except Nonverbal ability correlated significantly with RT, with Literacy showing the highest correlation, followed by Vocabulary, Working memory, Motor speed and Phonological awareness.

**Table 2:** **Correlations among the predictor variables, and between the predictor variables and naming RTs for 7-letter nonwords in block 1 of day 1**.

**Variable**	**1 Vocab**	**2 Literacy**	**3 Phon**	**4 Wkg mem**	**5 Nonverb**	**6 Mot**	**7 RT**
1. Vocabulary	–						
2. Literacy	0.656^**^						
3. Phonological awareness	−0.403^**^	−0.571^**^	–				
4. Working memory	0.266^*^	0.520^**^	0.432^**^	–			
5. Nonverbal ability	−0.014	−0.127	−0.175	−0.247	–		
6. Motor speed	−0.319^*^	−0.452^**^	−0.336^**^	−0.418^**^	0.149	–	
7. Block 1, 7-letter RTs	−0.584^**^	−0.739^**^	−0.377^**^	−0.444^**^	0.001	0.409^**^	–

* *p < 0.05*,

** p < 0.01. Note that phonological awareness was reverse transformed (thereby reversing the normal direction of correlations). Nonverbal ability and motor speed were square root transformed. RT was log transformed.

Linear mixed effects modeling was used to explore the ability of Vocabulary, Literacy, Phonological awareness, Working memory, Nonverbal ability and Motor speed to predict initial nonword reading speed and novel word learning. Linear mixed effects (LME) methods analyze all the available data and do not rely on averaging across participants or across items. They are particularly useful for analysing data from heterogeneous groups (such as individuals with dyslexia) because they allow differences in the baseline performance among participants and items (*random effects*) to be separated from the effects of the predictor variables (*fixed effects*) (Baayen et al., 2008; Jones et al., 2008). The analyses were conducted in R using the lme4 (Bates et al., 2012) and languageR (Baayen, 2009) packages.

#### Predicting initial nonword reading speed

The contribution of each predictor variable to predicting RTs for 7-letter nonwords presented in block 1 of day 1 was evaluated by using likelihood ratio tests to compare a model that contained all the fixed and random effects with a sequence of models in which different predictor variables were removed one at a time. These analyses showed that Literacy made a significant independent contribution to predicting nonword naming speed, χ^2^_(10)_ = 16.12, *p* < 0.001; β = −0.005, *t* = −4.30, *p* < 0.001. In contrast, Vocabulary, χ^2^_(10)_ = 2.71, *p* = 0.096, Phonological awareness, χ^2^_(10)_ = 1.41, *p* = 0.235, Working memory, χ^2^_(10)_ = 1.53, *p* = 0.217, Nonverbal ability, χ^2^_(10)_ = 1.37, *p* = 0.243, and Motor speed, χ^2^_(10)_ = 1.12, *p* = 0.293, made no independent contributions.

#### Predicting learning

Novel word learning was assessed in terms of the change in naming RTs for 7-letter nonwords between blocks 1 and 10 of day 1. RTs from both blocks were entered into the analysis. A categorical variable of Time was created to reflect the change in RTs between blocks 1 and 10. A set of predictor variables were then created which were the interactions involving Time with Vocabulary, Literacy, Phonological awareness, Working memory, Nonverbal ability and Motor speed. This makes it possible to evaluate the contribution of each independent variable to predict change in naming RTs to the 7-letter nonwords across blocks (Shek and Ma, 2011; Field, 2012). The effect of the categorical variable of Time was significant, χ^2^_(11)_ = 516.29, *p* < 0.001, reflecting the reduction in RTs from block 1 to block 10. The interactions of Time with Vocabulary, χ^2^_(17)_ = 6.57, *p* < 0.05; β = 0.002, *t* = 2.57, *p* < 0.05, and Time with Working memory, χ^2^_(17)_ = 26.12, *p* < 0.001; β = 0.003, *t* = 5.14, *p* < 0.001, were also significant. The interactions of Time with Literacy, χ^2^_(17)_ = 0.71, *p* = 0.401, Phonological awareness, χ^2^_(17)_ = 1.79, *p* = 0.181, Nonverbal ability, χ^2^_(17)_ = 3.65, *p* = 0.100, and Motor skill, χ^2^_(17)_ = 0.10, *p* = 0.753, made no independent contributions to predicting RT change across blocks.

In sum, reading latencies for the more difficult, 7-letter nonwords seen for the first time correlated significantly with all of the predictor variables except Nonverbal ability. The highest correlation was with Literacy. When the ability of each of the variables to predict naming RT was assessed in the context of the other variables (in analyses which took into account the differences between participants and items in overall naming speed), only Literacy was significant. Novel word learning was assessed as the change in RTs for 7-letter nonwords between blocks 1 and 10 of day 1. Only Vocabulary and Working memory predicted the degree of learning across blocks in session 1.

## Discussion

The adult dyslexics in the current experiment were all studying at university or in a college of higher education. They performed at a comparable level to typically-reading controls on a test of nonverbal ability (matrix reasoning) but had lower vocabulary scores, slower and less accurate reading and spelling of words, less efficient reading of nonwords, poorer phonological awareness, poorer performance on both verbal and nonverbal tests of span and working memory, and slower motor speed. These findings match other reports in the literature that dyslexics in higher education have cognitive problems that extend beyond reading and writing to wider aspects of linguistic, working memory and motor performance while typically sparing nonverbal reasoning (cf. Bruck, 1992; Gallagher et al., 1996; Snowling et al., 1997; Hatcher et al., 2002; Smith-Spark et al., 2003; Smith-Spark and Fisk, 2007; Callens et al., 2012; Warmington et al., 2013b). The working memory problems extend to visuospatial as well as verbal tasks (cf. Smith-Spark and Fisk, 2007; Menghini et al., 2011; Hachmann et al., 2014).

The largest difference between dyslexics and controls in the present study (as indicated by the effect size) was on the TOWRE Phonemic Decoding Efficiency test (Torgesen et al., 1999), a test of nonword reading. A great deal of effort is put into teaching phonic decoding skills to dyslexic children in the UK (Rose, 2009). The dyslexics who participated in our study had mastered the letter-sound correspondences of English sufficiently to enable them to read correctly nonwords like *drentcy* and *larquof* on the first encounter, but they were substantially slower than the controls. The results of the TOWRE-PDE indicate that pronouncing unfamiliar nonwords (and, by extension, unfamiliar real words) remains a problem for dyslexics in higher education (cf. Bruck, 1990; Ben-Dror et al., 1991; Reid et al., 2006; Wolff, 2009).

In the experimental task, the typical readers behaved very similarly to the participants in Maloney et al. (2009) who were drawn from a similar population. Letter length exerted a major effect on reading speeds for nonwords seen for the first time, but the impact of length declined as naming latencies reduced across blocks, becoming nonsignificant from block 4 of day 1. The results showed, therefore, that skilled adult readers can create representations of unfamiliar letter sequences after 4 or 5 presentations that allow them to recognize and pronounce the novel “words” quickly and to process their component letters in parallel.

The dyslexics were substantially slower at reading the nonwords throughout both sessions of the experiment. When the dyslexics read the 7-letter nonwords for the first time in block 1 of day 1, they did so with a mean latency that was over 300 ms slower than the controls. When performance on the 4- and 7-letter nonwords was compared, the dyslexics required 57 ms per letter in order to pronounce a nonword seen for the first time where the controls required just 23 ms per letter (less than half as much as the dyslexics). Ability at reading and spelling real words (“literacy”) predicted decoding speed across the two groups. When the effect of literacy was taken into account there was no additional effect of vocabulary, phonological awareness or working memory on decoding speed for these particular readers.

The dyslexics in the present study were clearly capable of visual word learning. Figure 1 shows that their naming latencies reduced across blocks and that their naming latencies to 4- and 7-letter nonwords eventually converged. Learning occurred considerably more slowly than in the dyslexics, however, than in the typical readers. Whereas the difference in RTs between shorter and longer nonwords became nonsignificant in the typical readers around the middle of session 1, the dyslexics showed slower naming of longer nonwords throughout session 1, only losing the length effect part-way into session 2 (day 7). The present study confirms, therefore, that the problems with word learning that have been documented in dyslexic children persist into early adulthood, even in high-functioning dyslexics (cf. Reitsma, 1983; Ehri and Saltmarsh, 1995; Mayringer and Wimmer, 2000; Share and Shalev, 2004; Elbro and Jensen, 2005; Thomson and Goswami, 2010; De Jong and Messbauer, 2011).

Importantly, the naming latencies for the dyslexics remained substantially longer than those of the typical readers through to the end of session 2. Figure 1 suggests that the difference between the two groups had more or less stabilized by the second half of session 2. We know that dyslexic university and college students read familiar words aloud more slowly than normal readers (Bruck, 1990; Ben-Dror et al., 1991; Reid et al., 2006; Wolff, 2009): one interpretation of that finding and the present evidence is that no amount of exposure to individual words will allow dyslexic students to reach the point where they can convert them from print to sound as efficiently as typical readers.

In terms of the DRC model of reading (Coltheart et al., 2001), less efficient reading of nonwords in the TOWRE-PDE test and in the experimental task indicates less efficient functioning of the nonlexical route in undergraduate dyslexics than in typical readers. Slower convergence between RTs to shorter and longer nonwords in the dyslexics suggest that the creation of new lexical entries in the orthographic input lexicon and the phonological output lexicon occurs less efficiently in adult dyslexics than typical readers. This results in a slower switch-over from sublexical to predominantly lexical reading in the dyslexics. Finally, the fact that nonword reading remains slower in the dyslexics than the controls even at the end of session two, combined with the fact that adult dyslexics are slower than controls to read familiar words aloud, indicates that the lexical route also functions less efficiently in adult dyslexics than in typical readers. That could be due to slower operation of the two lexicons or the pathways between them, or it could also be due to less efficient functioning of the final stages involving activating phoneme sequences and converting those sequences into articulation. Problems at the phonological output stage in dyslexics that compromise the functioning of both the lexical and nonlexical routes would be compatible with other evidence for impairments in dyslexics at the speech output stage (see Coltheart, 2005; Ziegler et al., 2008; Hawelka et al., 2010, for discussions of developmental dyslexia within a DRC framework).

Across the two groups, the ability to learn novel words (measured here as the change in RTs to longer nonwords between blocks 1 and 10 of day 1) was predicted by vocabulary and working memory. Ricketts et al. (2007) found that vocabulary predicted the ability of normal 8–10-year-olds to read words with irregular or exceptional spellings but did not predict their ability to read nonwords. By definition, irregular words like *deaf* or *yacht* violate the grapheme-phoneme correspondences of English. Nonlexical procedures cannot read those words correctly: readers must rely instead on word-specific learning and the creation of lexical entries. The results of Ricketts et al. (2007) are therefore in line with the present findings, albeit for a younger group of readers.

If a reader has a larger vocabulary, novel words they encounter in reading are likely to have more orthographic and phonological neighbors; that is, familiar words that look and sound like the novel words, differing from them by only a few letters or phonemes. Storkel et al. (2006) taught adults novel spoken words paired with novel objects through stories and pictures. Learning was better for nonwords with many neighbors than for nonwords with few neighbors. In the DRC model, words that are already established in the orthographic and phonological lexicons support the processing of new words or nonwords which resemble them. This is done through interactions between the two lexicons and the systems that encode and represent letter and phoneme sequences. Those interactions allow the model to process nonwords with many neighbors more efficiently than nonwords with fewer neighbors. Lexical support for novel words during learning could explain the advantage for nonwords with many neighbors reported by Storkel et al. (2006) and the benefit of a larger vocabulary found by Ricketts et al. (2007) and in the present study.

As regards the contribution of working memory, we noted in the Introduction that studies of children and young adults by Jarrold et al. (2009), Majerus and Boukebza (2013) and Martin and Ellis (2012) found a relationship between working memory and the ability to learn novel words, with working memory apparently related more closely to acquiring new word-forms rather than their meanings. Those observations fit well with the present findings. The DRC model does not engage with the working memory literature directly, but an important part of working memory is the interaction between short- and long-term memory systems exemplified by the interaction between phoneme representations and lexical entries (the phonological output lexicon in the DRC model). Jarrold et al. (2009) and Martin and Ellis (2012) explained the relationship they observed between verbal short-term memory and word learning in terms of individual differences in the ability to maintain accurate phonological representations of novel words. Majerus et al. (2006) argued that maintaining information about the order of phonemes in words is particularly important for successful word learning. In that context, we note the report by Hachmann et al. (2014) that short-term recall of order information is particularly impaired in dyslexia, which may contribute to their word learning problems.

Phonological awareness did not emerge as a predictor of either initial naming RTs or learning when the contributions of the other predictors were taken into account. Research has established that phonological awareness alone is not enough to improve decoding skills: only when phonological training is combined with training on the mappings between letters and phonemes does reading improve (Hatcher et al., 1994; Melby-Lervåg et al., 2012). Knowledge of the links between letters and sounds may be better captured by the kind of measures of word reading and spelling that went into the Literacy variable in the present study than by phonological awareness based on spoken stimuli and responses.

In conclusion, our results show that adult dyslexics in the UK university and further education system continue to experience difficulty reading novel words and nonwords. They are slower to read nonwords aloud than typical readers, requiring more time per letter to pronounce unfamiliar sequences of letters. They show learning of novel words as a result of repeated exposures, but they require more exposures than typical readers before they establish effective lexical representations. Even after multiple presentations their speed of reading aloud is substantially slower than typical readers. They remain slower than typical readers even at reading familiar words aloud. Across both dyslexic and typical readers, decoding speed for nonwords was predicted by skill at reading and spelling real words (“literacy”) while individual differences in word learning were predicted by vocabulary size and working memory. As others have also shown, the problems that adult dyslexics experience extend beyond reading and spelling to word learning, vocabulary, phonological awareness, working memory and even basic motor speed. Taken together, those problems will conspire to make it very challenging for adult dyslexics to function successfully within higher education.

### Conflict of interest statement

The authors declare that the research was conducted in the absence of any commercial or financial relationships that could be construed as a potential conflict of interest.
