# Supplementary material for: Visual word learning in adults with dyslexia
Source: Front Hum Neurosci. 2014 May 6;8:264. doi: 10.3389/fnhum.2014.00264 (PMC4018562; doi:10.3389/fnhum.2014.00264)
Supplement: Supplementary file 1 [file Appendix.DOCX]

**APPENDIX** **|** **Statistical analysis of the naming RT data.**

| ***Effects*** | ***By-participants analysis (F1)*** |
| --- | --- |
| **Days 1 and 7 combined (global analysis)** | |
| Group | *F_1_*(1, 58) = 25.81, *MSE* = 9291901, *p* < .001, *η*^2^_p_ = .308 |
| Day | *F_1_*(1, 58) = 71.39, *MSE* = 3216320, *p* < .001, *η*^2^_p_ = .552 |
| Blocks | *F_1_*(2.67, 154.61) = 63.56, *MSE* = 1389879, *p* < .001, *η*^2^_p_ = .523 |
| Length | *F_1_*(1, 58) = 84.80, *MSE* = 572957, *p* < .001, *η*^2^_p_ = .594 |
| Group x Day | *F_1_*(1, 58) = 10.54, *MSE* = 474883, *p* < .005, *η*^2^_p_ = .154 |
| Group x Blocks | *F_1_*(9, 522) = 10.28, *MSE* = 66551, *p* < .001, *η*^2^_p_ = .150 |
| Group x Length | *F_1_*(1, 58) = 17.89, *MSE* = 120898, *p* < .001, *η*^2^_p_ = .236 |
| Day x Blocks | *F_1_*(4.48, 259.85) = 16.88, *MSE* = 132020, *p* < .001, *η*^2^_p_ = .225 |
| Day x Length | *F_1_*(1, 58) = 83.35, *MSE* = 130627, *p* < .001, *η*^2^_p_ = .590 |
| Blocks x Length | *F_1_*(5.99, 347.46) = 24.32, *MSE* = 43728, *p* < .001, *η*^2^_p_ = .295 |
| Group x Day x Blocks | *F_1_*(9, 522) = 4.86, *MSE* = 18911, *p* < .001, *η*^2^_p_ = .077 |
| Group x Day x Length | *F_1_*(1, 58) = 20.46, *MSE* = 32072, *p* < .001, *η*^2^_p_ = .261 |
| Group x Blocks x Length | *F_1_*(9, 522) = 4.31, *MSE* = 5155, *p* < .001, *η*^2^_p_ = .069 |
| Day x Blocks x Length | *F_1_*(5.94, 344.38) = 4.21, *MSE* = 7313, *p* < .001, *η*^2^_p_ = .068 |
| Group x Day x Blocks x Length | *F_1_*(9, 522) = 1.97, *MSE* = 2258, *p* < .05, *η*^2^_p_ = .033 |
|  |  |
| **Day 1 only** |  |
| Group | *F_1_*(1, 58) = 30.83, *MSE* = 6984003, *p* < .001, *η*^2^_p_ = .347 |
| Blocks | *F_1_*(3.11, 180.35) = 59.81, *MSE* = 1152122, *p* < .001, *η*^2^_p_ = .508 |
| Length | *F_1_*(1, 58) = 103.02, *MSE* = 625368, *p* < .001, *η*^2^_p_ = .640 |
| Group x Blocks | *F_1_*(9, 522) = 11.28, *MSE* = 75041, *p* < .001, *η*^2^_p_ = .163 |
| Group x Length | *F_1_*(1, 58) = 22.86, *MSE* = 138753, *p* < .001, *η*^2^_p_ = .283 |
| Blocks x Length | *F_1_*(5.41, 313.79) = 19.84, *MSE* = 46346, *p* < .001, *η*^2^_p_ = .255 |
| Group x Blocks x Length | *F_1_*(9, 522) = 4.07, *MSE* = 5711, *p* < .001, *η*^2^_p_ = .066 |
|  |  |
| ***Day 1 typical readers only*** |  |
| Blocks | *F_1_*(2.98, 86.37) = 19.55, *MSE* = 203021, *p* < .001, *η*^2^_p_ = .403 |
| Length | *F_1_*(1, 29) = 19.40, *MSE* = 87490, *p* < .001, *η*^2^_p_ = .401 |
| *Bonferroni-corrected t-tests (*α *= .005) comparing RTs to 4- and 7-letter nonwords in each block of day 1* | |
| Block 1 | *t_1_*(29) = 6.38, *p* < *.*001 |
| Block 2 | *t_1_*(29) = 4.57, *p* < *.*001 |
| Block 3 | *t_1_*(29) = 5.30, *p* < *.*001 |
| Block 4 | *t_1_*(29) = 1.39, *p = .*175 |
| Block 5 | *t_1_*(29) = 2.67, *p = .*012 |
| Block 6 | *t_1_*(29) = 0.84, *p = .*410 |
| Block 7 | *t_1_*(29) = 2.05, *p = .*049 |
| Block 8 | *t_1_*(29) = 1.29, *p = .*209 |
| Block 9 | *t_1_*(29) = 1.90, *p = .*068 |
| Block 10 | *t_1_*(29) = 1.28, *p = .*211 |
|  |  |
| ***Day 1 dyslexics only*** |  |
| Blocks | *F_1_*(2.94, 85.15) = 41.11, *MSE* = 1244155, *p* < .001, *η*^2^_p_ = .586 |
| Length | *F_1_*(1, 29) = 88.68, *MSE* = 676631, *p* < .001, *η*^2^_p_ = .754 |
| *Bonferroni-corrected t-tests (*α *= .005) comparing RTs to 4- and 7-letter nonwords in each block of day 1* | |
| Block 1 | *t_1_*(29) = 8.47, *p* < *.*001 |
| Block 2 | *t_1_*(29) = 6.45, *p* < *.*001 |
| Block 3 | *t_1_*(29) = 5.73, *p* < *.*001 |
| Block 4 | *t_1_*(29) = 4.81, *p* < *.*001 |
| Block 5 | *t_1_*(29) = 4.21, *p* < *.*001 |
| Block 6 | *t_1_*(29) = 2.89, *p = .*007 |
| Block 7 | *t_1_*(29) = 5.34, *p* < *.*001 |
| Block 8 | *t_1_*(29) = 2.87, *p = .*008 |
| Block 9 | *t_1_*(29) = 4.29, *p* < *.*001 |
| Block 10 | *t_1_*(29) = 4.29, *p* < *.*001 |
|  |  |
| **Day 7 only** |  |
| Group | *F_1_*(1, 58) = 15.58, *MSE* = 2782781, *p* < .001, *η*^2^_p_ = .212 |
| Blocks | *F_1_*(3.33, 193.05) = 21.35, *MSE* = 214521, *p* < .001, *η*^2^_p_ = .269 |
| Length | *F_1_*(1, 58) = 34.71, *MSE* = 78216, *p* < .001, *η*^2^_p_ = .374 |
| Group x Blocks | *F_1_*(9, 522) = 2.81, *MSE* = 10421, *p* < .005, *η*^2^_p_ = .046 |
| Group x Length | *F_1_*(1, 58) = 6.31, *MSE* = 14216, *p* < .05, *η*^2^_p_ = .098 |
| Blocks x Length | *F_1_*(9, 522) = 6.46, *MSE* = 6071, *p* < .001, *η*^2^_p_ = .100 |
| Group x Blocks x Length | *F_1_*(9, 522) = 1.81, *MSE* = 1701, *p* = .064, *η*^2^_p_ = .030 |
|  |  |
|  |  |
| ***Day 7 typical readers only*** |  |
| Blocks | *F_1_*(3.24, 93.85) = 8.42, *MSE* = 46950, *p* < .001, *η*^2^_p_ = .225 |
| Length | *F_1_*(1, 29) = 7.52, *MSE* = 12871, *p* = .01, *η*^2^_p_ = .206 |
| *Bonferroni-corrected t-tests (*α *= .005) comparing RTs to 4- and 7-letter nonwords in each block of day 7* | |
| Block 1 | *t_1_*(29) = 4.68, *p* < *.*001 |
| Block 2 | *t_1_*(29) = 1.33, *p = .*193 |
| Block 3 | *t_1_*(29) = 0.55, *p = .*589 |
| Block 4 | *t_1_*(29) = 1.68, *p = .*104 |
| Block 5 | *t_1_*(29) = 0.28, *p = .*786 |
| Block 6 | *t_1_*(29) = 0.15, *p = .*883 |
| Block 7 | *t_1_*(29) = 0.02, *p = .*982 |
| Block 8 | *t_1_*(29) = 2.00, *p = .*055 |
| Block 9 | *t_1_*(29) = 1.74, *p = .*093 |
| Block 10 | *t_1_*(29) = 0.71, *p = .*482 |
|  |  |
| ***Day 7 dyslexics only*** |  |
| Blocks | *F_1_*(2.84, 82.46) = 13.43, *MSE* = 230665, *p* < .001, *η*^2^_p_ = .317 |
| Length | *F_1_*(1, 29) = 28.47, *MSE* = 79562, *p* < .001, *η*^2^_p_ = .495 |
| *Bonferroni-corrected t-tests (*α *= .005) comparing RTs to 4- and 7-letter nonwords in each block of day 7* | |
| Block 1 | *t_1_*(29) = 5.53, *p* < *.*001 |
| Block 2 | *t_1_*(29) = 3.93, *p* < *.*001 |
| Block 3 | *t_1_*(29) = 3.02, *p = .*005 |
| Block 4 | *t_1_*(29) = 0.58, *p = .*568 |
| Block 5 | *t_1_*(29) = 1.65, *p = .*110 |
| Block 6 | *t_1_*(29) = 0.46, *p = .*648 |
| Block 7 | *t_1_*(29) = 2.43, *p = .*021 |
| Block 8 | *t_1_*(29) = 1.93, *p = .*063 |
| Block 9 | *t_1_*(29) = 2.28, *p = .*030 |
| Block 10 | *t_1_*(29) = 1.35, *p = .*188 |
|  |  |
