# Supplementary material for: Visual word learning in adults with dyslexia
Source: Front Hum Neurosci. 2014 May 6;8:264. doi: 10.3389/fnhum.2014.00264 (PMC4018562; doi:10.3389/fnhum.2014.00264)
Supplement: Supplementary file 2 [file Table2.PDF]

Table S1 | Mean latencies of correct, trimmed responses, standard deviations (SS), and per cent correct responses for 4- and 7-letter nonwords in blocks 1 to 10 of day 1 and day 7 in dyslexics and typical readers.

	Day 1									
**Blocks**	1	2	3	4	5	6	7	8	9	10
**Dyslexic readers**										
4-letter nonwords										
Mean RT	803	729	701	650	646	636	639	629	606	613
SD	180	172	151	125	137	115	106	111	117	111
% correct	100.0	98.9	98.6	98.3	97.2	98.1	98.9	97.2	98.1	97.5
7-letter nonwords										
Mean RT	975	838	765	719	702	670	689	660	654	649
SD	226	193	161	154	151	132	134	127	117	118
% correct	98.3	98.9	95.6	96.9	96.9	96.4	96.1	97.8	97.5	98.1
**Typical readers**										
4-letter nonwords										
Mean RT	597	551	529	537	530	530	525	520	512	511
SD	91	85	71	89	92	111	93	84	83	109
% correct	99.7	96.9	98.1	98.3	97.8	98.1	96.4	96.9	96.9	98.1
7-letter nonwords										
Mean RT	666	585	568	548	552	539	541	530	528	526
SD	126	102	101	99	104	113	93	77	93	113
% correct	95.6	96.9	98.6	96.7	97.5	97.2	98.1	97.8	98.6	98.1

	Day 7									
**Blocks**	1	2	3	4	5	6	7	8	9	10
**Dyslexic readers**										
4-letter nonwords										
Mean RT	657	589	586	594	575	587	565	573	572	561
SD	166	130	144	151	101	119	101	99	93	106
% correct	98.3	98.1	98.3	97.8	98.6	98.3	96.9	97.5	98.9	98.1
7-letter nonwords										
Mean RT	721	635	618	599	593	582	585	589	593	574
SD	167	142	133	144	108	96	108	113	100	104
% correct	97.8	98.1	97.8	97.5	96.9	96.7	96.9	96.9	95.8	96.9
**Typical readers**										
4-letter nonwords										
Mean RT	525	508	505	491	496	498	492	481	486	484
SD	87	85	90	73	79	83	87	85	93	82
% correct	98.3	99.2	98.3	96.9	98.6	98.3	98.1	97.8	98.1	99.4
7-letter nonwords										
Mean RT	562	515	509	503	497	498	492	494	498	488
SD	104	82	100	70	66	76	80	77	81	89
% correct	98.3	97.5	97.8	98.6	98.1	96.4	97.5	98.1	97.8	98.1
